# Making the Invisible Visible: Advanced Neuroimaging Techniques in Focal Epilepsy

**DOI:** 10.3389/fnins.2021.699176

**Published:** 2021-07-27

**Authors:** Daichi Sone

**Affiliations:** ^1^Department of Psychiatry, The Jikei University School of Medicine, Tokyo, Japan; ^2^Department of Clinical and Experimental Epilepsy, UCL Institute of Neurology, London, United Kingdom

**Keywords:** focal epilepsy, magnetic resonance imaging, advanced neuroimaging, structural neuroimaging, diffusion neuroimaging, functional neuroimaging

## Abstract

It has been a clinically important, long-standing challenge to accurately localize epileptogenic focus in drug-resistant focal epilepsy because more intensive intervention to the detected focus, including resection neurosurgery, can provide significant seizure reduction. In addition to neurophysiological examinations, neuroimaging plays a crucial role in the detection of focus by providing morphological and neuroanatomical information. On the other hand, epileptogenic lesions in the brain may sometimes show only subtle or even invisible abnormalities on conventional MRI sequences, and thus, efforts have been made for better visualization and improved detection of the focus lesions. Recent advance in neuroimaging has been attracting attention because of the potentials to better visualize the epileptogenic lesions as well as provide novel information about the pathophysiology of epilepsy. While the progress of newer neuroimaging techniques, including the non-Gaussian diffusion model and arterial spin labeling, could non-invasively detect decreased neurite parameters or hypoperfusion within the focus lesions, advances in analytic technology may also provide usefulness for both focus detection and understanding of epilepsy. There has been an increasing number of clinical and experimental applications of machine learning and network analysis in the field of epilepsy. This review article will shed light on recent advances in neuroimaging for focal epilepsy, including both technical progress of images and newer analytical methodologies and discuss about the potential usefulness in clinical practice.

## Introduction

Epilepsy is a common chronic brain disease, which affects around 50 million people all over the world (Leonardi and Ustun, 2002; GBD 2016 Epilepsy Collaborators, 2019). The burden of epilepsy includes recurrent seizures, their physical and psychosocial problems, and various comorbidities (GBD 2016 Epilepsy Collaborators, 2019). While seizures can be controlled by anti-seizure medicine in over 60% of patients with epilepsy (Kwan and Brodie, 2000; Chen et al., 2018b), the rest of them experience drug-resistant seizures, which may result in poorer quality of life (Kubota and Awaya, 2010). Epilepsy surgery is a well-established option to remediate patients with drug-resistant epilepsy, and particularly accurate localization of epileptogenic focus has a key role for the successful surgical resection in focal epilepsy (Rathore and Radhakrishnan, 2015).

Neuroimaging is an essential examination for epilepsy, and one of its major roles is to visualize epileptogenic lesions, particularly in patients with drug-resistant focal seizures (Bernasconi et al., 2019). However, a part of cases with focal epilepsy show visually normal MRI, which is called “MRI-negative” epilepsy (So and Lee, 2014), and the proportion of MRI-negative cases was supposed to be up to 30% in temporal lobe epilepsy (Muhlhofer et al., 2017). Since unsuccessful localization of focus by MRI may lead to poorer surgical seizure outcome (So and Lee, 2014), accurate visualization of epileptogenic lesions by neuroimaging techniques has been a long-standing challenge in epilepsy.

Thus, the current review will shed light on recent advanced neuroimaging techniques for focus detection as well as conventional standard and quantitative analysis.

## Conventionally “Visible” Structural Lesions

**FIGURE 1:**
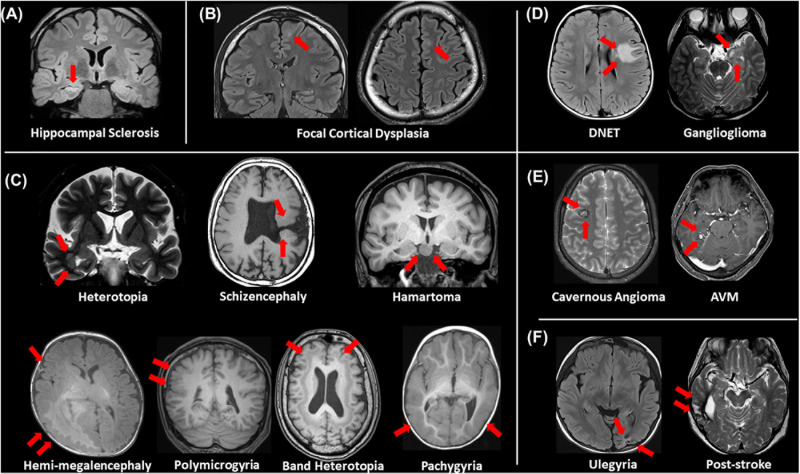
An overview of visible epileptogenic lesions (red arrows). **(A)** Hippocampal sclerosis, **(B)** focal cortical dysplasia, **(C)** other malformations of cortical development, **(D)** neoplasms, **(E)** vascular malformations, and **(F)** cerebrovascular lesions.

**FIGURE 2:**
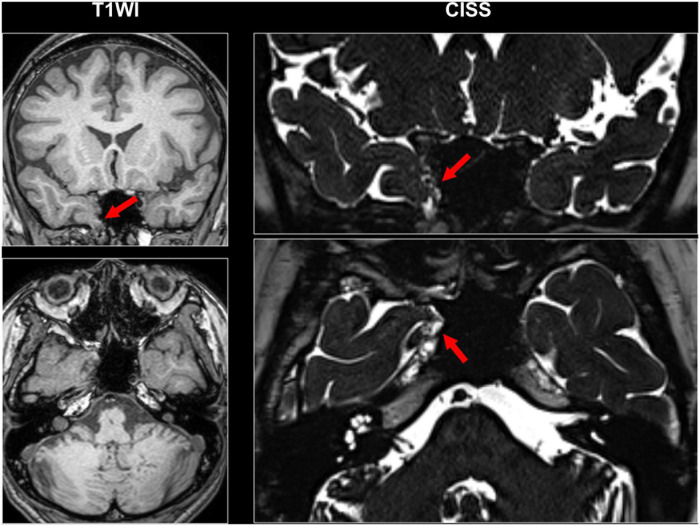
A case with drug-resistant temporal lobe epilepsy and encephalocele. Constructive interference in steady-state (CISS) imaging was helpful for detection by enhancing the contrast between brain parenchyma and cerebrospinal fluid.

Even though a lot of quantitative methodologies have been developed, visual inspection is still an important and standard approach for focus detection. Figure 1 presents an overview of conventionally visible epileptogenic lesions, including hippocampal sclerosis, focal cortical dysplasia and other malformation of cortical development, neoplasms, vascular malformations, and cerebrovascular diseases. Before discussing about MRI-negative epilepsy, epileptologists should be aware of these common epileptogenic lesions. Particularly, the two common etiologies, i.e., hippocampal sclerosis and focal cortical dysplasia, may need careful and specific attention for detection, as only subtle abnormalities may sometimes be found (Bernasconi et al., 2019). Additionally, meningoencephalocele has been recently recognized as another etiology in drug-resistant focal epilepsy, which may sometimes show only subtle abnormalities (Saavalainen et al., 2015; Tse et al., 2020). In cases with encephalocele, constructive interference in steady-state (CISS) imaging may be helpful for detection by enhancing the contrast between brain parenchyma and cerebrospinal fluid (Wang et al., 2017) (Figure 2). On the other hand, we need to keep in mind that the detected abnormalities may not always cause the seizures, in cases with incidental lesions.

**FIGURE 3:**
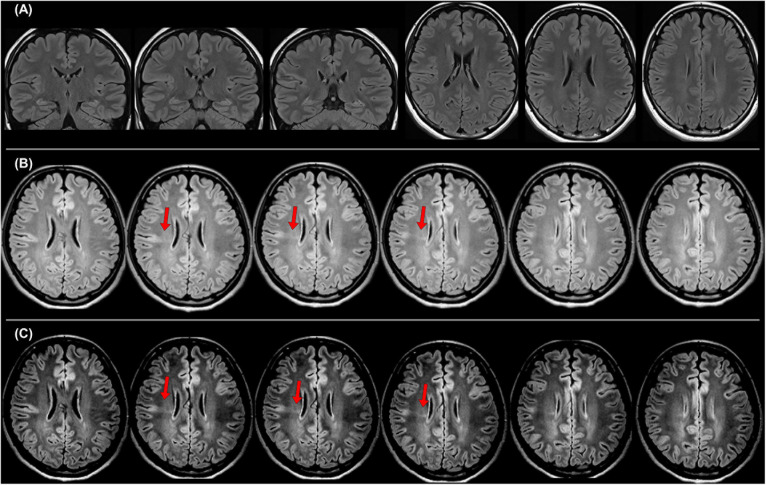
A case with drug-resistant focal epilepsy, who benefited from the official standard protocol for epilepsy. It was impossible to detect any abnormalities in both coronal and axial 2D fluid-attenuated inversion recovery (FLAIR) images with 3-mm slice thickness **(A)**, but the 3D FLAIR images revealed findings of a bottom-of-sulcus-type focal cortical dysplasia with transmantle sign **(B)**, and changing the signal range is sometimes helpful to clearly visualize the lesion **(C)**. The pathological finding was focal cortical dysplasia type IIb.

**FIGURE 4:**
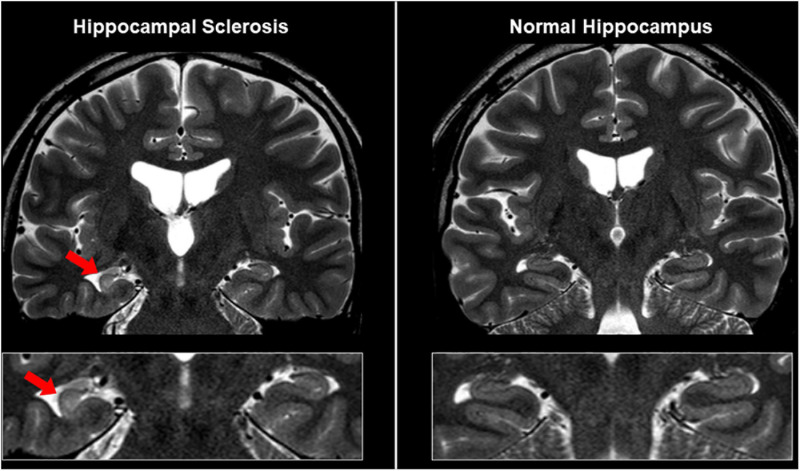
MRI findings in a case with unilateral hippocampal sclerosis (left). The affected hippocampus showed hippocampal atrophy and T2 hyperintensity, and thinning and blurring of the molecular layer, compared with the contralateral side or normal case (right).

It is also important to differentiate epileptogenic lesions, particularly focal cortical dysplasia, from other findings, such as unspecific aging-related changes showing T2 hyperintensity. For that, we need to consider the main features of focal cortical dysplasia, including cortical thickening, blurring of gray–white matter junction, cortical or white matter T2 hyperintensity, and transmantle sign (De Vito et al., 2021) (Figures 1B, 3). To detect hippocampal sclerosis, which is the most common etiology of temporal lobe epilepsy (Thom, 2014), attention should be paid to hippocampal atrophy and T2 hyperintensity, and thinning and blurring of the molecular layer (Bernasconi et al., 2019; De Vito et al., 2021) (Figure 4). As described, epileptogenic lesions are sometimes subtle, and 3D acquisition with reformats is important (De Vito et al., 2021). Therefore, we should be careful about motion artifact and quality control.

## Recommendation of the Official Standard Protocol for Epilepsy

In 2019, the International League Against Epilepsy (ILAE) published the official recommendation of structural MRI for epilepsy (Bernasconi et al., 2019). In that, the following protocols were recommended as a standard: 3D millimetric T1-weighted images (T1WI) and fluid-attenuated inversion recovery (FLAIR) images, and 2D submillimetric coronal T2-weighted images (T2WI). Figure 3 shows a representative case with drug-resistant focal epilepsy, who benefited from 3D millimetric FLAIR images. It was impossible to detect any abnormalities in both coronal and axial 2D FLAIR images with 3-mm slice thickness (Figure 3A), but the 3D FLAIR images revealed findings of a bottom-of-sulcus-type focal cortical dysplasia with transmantle sign (Figure 3B), and changing the signal range may sometimes be helpful to clearly visualize the lesion (Figure 3C). The patient underwent surgical resection, and the pathological result was focal cortical dysplasia type IIb. Thus, the optimal MRI protocol for epilepsy may be able to make the previously invisible lesions visible.

However, even with such optimized protocols, we sometimes encounter patients with visually normal MRI. To detect the conventionally invisible epileptogenic lesions, efforts have been made to seek for useful advanced neuroimaging techniques in drug-resistant focal epilepsy (Bernasconi and Wang, 2021).

## Advanced Structural Imaging

**FIGURE 5:**
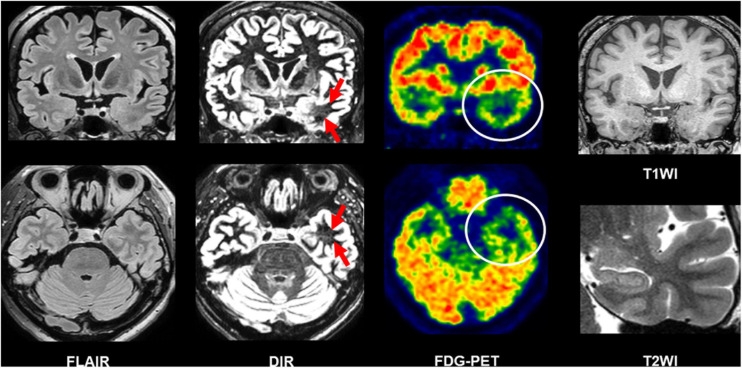
A case of conventionally MRI-negative PET-positive temporal lobe epilepsy (TLE). While it was difficult to detect abnormalities on FLAIR, double inversion recovery (DIR) visualized hyperintensity within the anterior temporal white matter of the focus side. T1-weighted images (T1WI) and T2WI were also intact including the hippocampus.

Beyond the recommended MRI protocol, newer structural MRI sequences have been suggested to provide additional usefulness. Double inversion recovery (DIR), which shows a high contrast between gray and white matters (Ryan, 2016), has been increasingly reported as a useful sequence to detect epileptogenic lesions in temporal lobe epilepsy (TLE) and extratemporal focal epilepsy (Li et al., 2011; Morimoto et al., 2013a, b; Granata et al., 2016; Wong-Kisiel et al., 2016; Wychowski et al., 2016; Sone et al., 2021). In TLE, the superiority of DIR to FLAIR for the detection of anterior temporal white matter abnormalities in the focus side in TLE was reported by both qualitative and quantitative evaluations (Morimoto et al., 2013a; Sone et al., 2021). Figure 5 describes a case of conventionally MRI-negative PET-positive unilateral TLE, in which increased DIR signals can be found in the focus side, while it was difficult to detect on FLAIR, T1WI, and T2WI. More recently, fluid and white matter suppression (FLAWS) has been reported for better visualization of focal cortical dysplasia even in conventionally MRI-negative cases (Chen et al., 2018a; Sun et al., 2021). FLAWS suppresses the white matter and cerebrospinal fluid signals and then generate gray matter-specific images (Tanner et al., 2012; Chen et al., 2018a). Thus, the enhanced contrast between gray and white matters by these newer sequences may improve the visualization of epileptic foci. In addition, edge-enhancing gradient echo (EDGE) imaging was reported to allow us to detect focal cortical dysplasia by directly visualizing the boundary between gray and white matters (Middlebrooks et al., 2020).

## Advanced Diffusion Imaging

**FIGURE 6:**
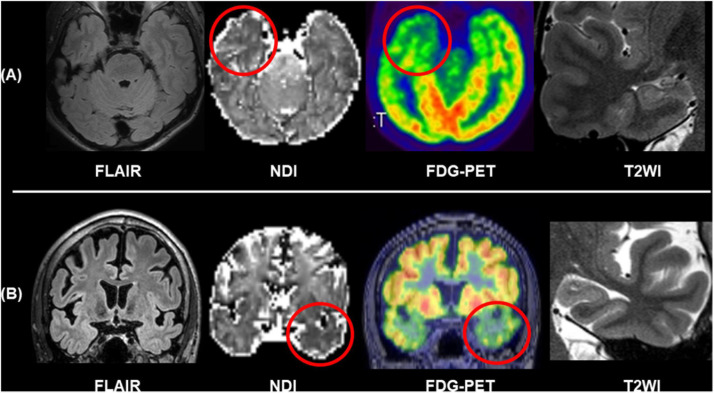
Two cases of conventionally MRI-negative PET-positive TLE. While no abnormalities were found in FLAIR and T2WI including the hippocampus, neurite orientation dispersion and density imaging (NODDI) revealed reduced neurite density of the focus side. **(A)** Modified from Sone et al. (2018). **(B)** Modified from Sone (2019).

The progress in diffusion MRI has been an emerging topic in the field of neurology and psychiatry. Particularly, multi-shell protocols of diffusion MRI, including diffusion kurtosis imaging (DKI), q-space imaging (QSI), restriction spectrum imaging (RSI), and neurite orientation dispersion and density imaging (NODDI), have provided further information on brain microstructures (Cohen and Assaf, 2002; Jensen et al., 2005; White et al., 2013; Sone, 2019). In the field of epilepsy, NODDI and RSI have been repeatedly reported for their usefulness (Winston et al., 2014; Loi et al., 2016; Reyes et al., 2018; Rostampour et al., 2018; Sone et al., 2018; Lorio et al., 2020; Winston et al., 2020; Shao et al., 2021). Neurite orientation dispersion and density imaging allows us to investigate neurite density and orientation dispersion of the brain microstructures, and reduced neurite density has been consistently found in visible focal cortical dysplasia (Winston et al., 2014; Lorio et al., 2020). Neurite orientation dispersion and density imaging may also visualize neurite abnormalities within the focus even in MRI-negative cases (Sone et al., 2018). Figure 6 represents two cases with conventionally MRI-negative PET-positive unilateral TLE, which showed reduced neurite density within the anterior temporal lobe of the focus side. In TLE with hippocampal sclerosis, reductions of neurite orientation dispersion as well as neurite density were reported (Sone et al., 2018). Additionally, NODDI could help in better visualization of cortical tubers in tuberous sclerosis (Shao et al., 2021). RSI is another advanced diffusion MRI using multi-shell, reduced neurite density, and its correlation with clinical symptoms in epilepsy was also confirmed by RSI (Loi et al., 2016; Reyes et al., 2018). Thus, advances in diffusion MRI may be a promising tool for patients with drug-resistant focal epilepsy and invisible lesions on conventional MRI.

## Advanced Functional Neuroimaging

**FIGURE 7:**
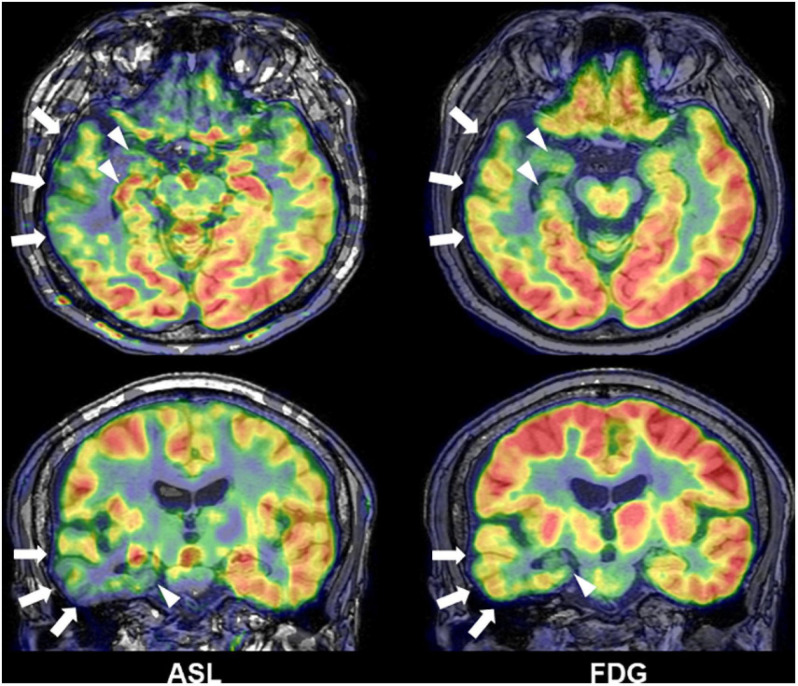
A case of temporal lobe epilepsy with hippocampal sclerosis. Both Arterial spin labeling (ASL) and 18F-FDG PET showed reduced signals around the temporal lobe of focus side (modified from Sone et al., 2019).

Interictal reduction of glucose metabolisms in ^18^F-FDG PET and ictal hyperperfusion detected by SPECT are traditional and established biomarkers for the detection of focus in drug-resistant epilepsy and often effective for MRI-negative cases (Kumar and Chugani, 2013; Shigemoto et al., 2020). In addition to nuclear imaging, recent advances in functional neuroimaging may further improve the detection of focus. Arterial spin labeling (ASL) is a non-invasive method to visualize brain perfusion by MRI (Haller et al., 2016) and, thus, expected to detect abnormal cerebral blood flow, particularly interictal reduction, around the epileptogenic foci in epilepsy (Figure 7) (Boscolo Galazzo et al., 2016; Shang et al., 2018; Wang et al., 2018; Sone et al., 2019; Lam et al., 2020). Although ASL might not surpass ^18^F-FDG PET in terms of detectability of focus (Sone et al., 2019), its non-invasive nature and wide availability will guarantee a supplemental role in clinical practice. Functional MRI triggered by electroencephalogram (EEG-fMRI) is another newer tool of functional imaging for focus detection. EEG-fMRI can non-invasively detect the hemodynamic signals related with interictal epileptic discharges on EEG (van Graan et al., 2015), and then it can be utilized to visualize the epileptogenic zone and its propagations (Khoo et al., 2017, 2018).

## Quantitative Analysis and Post-Processing

Another solution for MRI-negative drug-resistant epilepsy is quantitative analysis and post-processing of images. It is known that quantitative hippocampal volumetry and signal analysis improve the visual detectability of hippocampal sclerosis (Coan et al., 2014), and better segmentation and detailed hippocampal profiling methods have also been developed (Winston et al., 2013; Vos et al., 2020). The Morphometric Analysis Program (MAP) is a well-investigated software to generate voxel-based morphometric maps, which can visualize subtle blurring of the gray–white boundary or abnormal cortical surface, using 3D T1WI. In fact, many studies confirmed the usefulness of MAP for the detection of focal cortical dysplasia (Kassubek et al., 2002; Huppertz et al., 2005; Wagner et al., 2011; Wang et al., 2015; Lin et al., 2018; Demerath et al., 2020) or band heterotopia (Huppertz et al., 2008). In addition to T1WI, usefulness of quantitative FLAIR or DIR analysis was also reported (Rugg-Gunn et al., 2006; Focke et al., 2009).

Machine learning is an emerging topic in this field; the advantage of machine-learning may include the accurate, automated, and fast pattern learning, which could be utilized to develop and/optimize clinical algorithms. Currently, studies on machine learning and epilepsy imaging reported its usefulness in the lateralization of TLE (Pustina et al., 2015; Bennett et al., 2019; Beheshti et al., 2020a, b) or automated detection of focal cortical dysplasia (Hong et al., 2014; Hong et al., 2016; Adler et al., 2017; Tan et al., 2018). While machine leaning has provided promising results for the detection of focus in epilepsy, we may need to develop and validate consistent methodology given the diversity of methods (Sone and Beheshti, 2021). Furthermore, network analysis is another trend in epilepsy (Bernhardt et al., 2015), and literature suggested that network metrics derived from neuroimaging could also be used for focus detection when combined with machine learning (Chiang et al., 2015; Yang et al., 2015; Kamiya et al., 2016; Fallahi et al., 2020).

## Multimodal Imaging

Combination of multimodal imaging is also important for precise localization of focus (Kurian et al., 2007). Concordance across different modalities supports successful epilepsy surgery (Rathore and Radhakrishnan, 2015), and in addition, coregistered images would improve visual detectability of epileptogenic foci, which was demonstrated by a study using MRI and ^18^F-FDG PET (Salamon et al., 2008). Multimodal imaging is also a topic in machine learning studies (Pustina et al., 2015; Bennett et al., 2019). Given the importance of multiple modalities in epilepsy, developing a platform for fusion of data (Marecek et al., 2021) would become a significant work for the future.

## Seven-Tesla MRI

Seven-tesla (7T) MRI is expected to yield improved detectability over 3T MRI, by the ultra-high-field magnetic strength (van Lanen et al., 2021). Despite the still limited access to 7T MRI, there have been several studies reporting its usefulness in epilepsy (De Ciantis et al., 2016; Veersema et al., 2017; Bartolini et al., 2019; Feldman et al., 2019). On the other hand, diagnostic gain of 7T over conventional MRI has been variable, ranging from 8 to 67% (van Lanen et al., 2021), and thus, further studies would be needed to establish the utility of 7T MRI for clinical use in patients with epilepsy.

## Establishment of Clinical MRI Standards for Epilepsy

While this review focused on the recent progress in newer imaging techniques, uniformity of the MRI protocols is of great relevance in clinical epileptology. To establish a practical standard, various aspects need to be considered, including magnetic field strength, imaging resolution, and acquisition time.

Regarding the magnetic field strength, 1.5- or 3-T MRI scanners are currently utilized in clinical practice. In principle, 3-T MRI provides a better signal-to-noise ratio and higher resolution of images, although we need to pay more careful attention to flow and motion artifact in 3-T scanners (Martinez-Rios et al., 2016; De Vito et al., 2021). Indeed, some previous studies reported better identification of epileptogenic lesions by 3- than by 1.5-T MRI, and the use of 3-T MRI may improve the clinical decision making (Knake et al., 2005; Zijlmans et al., 2009; Mellerio et al., 2014; Rubinger et al., 2016). The imaging resolution should be along with the official recommendation of ILAE (Bernasconi et al., 2019), i.e., 3D isotropic T1WI and FLAIR images with millimetric voxels (1 × 1 × 1 mm^3^), and 2D submillimetric T2WI designed for hippocampal evaluation. More advanced techniques, which were reviewed in this article, may be considered as additional imaging. On the other hand, however, such additions usually require longer acquisition time, which may become a trade-off dilemma for clinically acceptable epilepsy imaging. Thus, those advanced imaging methods need to become more established, particularly by robustly revealing the clinical usefulness, e.g., long-term prognosis of surgery. The manufacturer of MRI scanners is another important factor for the uniformity of epilepsy protocols, as some newer sequences have been developed by each specific manufacturer.

## Limitation and Future Challenge

As noted above, compared with conventionally established sequences, the usefulness of advanced imaging still needs to be more robustly elucidated. Although most studies reported potentials of better focus detection, long-term seizure outcomes after resection of the abnormal areas are rarely investigated, so far. Additionally, the cost effectiveness of acquisition time should be kept in mind. Thus, future studies should include more comprehensive and robust comparisons between imaging modalities and clinical parameters, as well as consideration of time efficiency. Another important topic in epilepsy imaging is the preclinical MRI studies to identify the underlying mechanism and time course of epileptogenesis (Immonen et al., 2019; Reddy et al., 2019). Advanced neuroimaging methods may provide further information for basic research on epilepsy. Eventually, in addition to focus detection, neuroimaging could contribute to elucidation of the neurobiological mechanisms, brain functions, and longitudinal brain changes in epilepsy (Galovic et al., 2019; Bernasconi and Wang, 2021). Thus, clinical and basic applications of advanced neuroimaging would be promising for better understanding and improved clinical practice for epilepsy.

## Conclusion

There have been various, continuous efforts to better visualize epileptogenic foci in drug-resistant focal epilepsy. The promising advances in structural, diffusion, and functional neuroimaging, as well as quantitative processing and machine learning, may provide critical information for epilepsy surgery and benefit patients with drug-resistant focal epilepsy.

## Author Contributions

DS was the sole author of this manuscript and contributed to all aspects.

## Conflict of Interest

The author declares that the research was conducted in the absence of any commercial or financial relationships that could be construed as a potential conflict of interest.

## Publisher’s Note

All claims expressed in this article are solely those of the authors and do not necessarily represent those of their affiliated organizations, or those of the publisher, the editors and the reviewers. Any product that may be evaluated in this article, or claim that may be made by its manufacturer, is not guaranteed or endorsed by the publisher.
